# Tsc22d3 promotes morphine tolerance in mice through the GPX4 ferroptosis pathway

**DOI:** 10.18632/aging.205903

**Published:** 2024-06-05

**Authors:** Yan Chen, Shan Li, Fenghui Guo

**Affiliations:** 1 Department of Anesthesiology, Children’s Hospital of Hebei Province, Shijiazhuang 050071, Hebei, P.R. China; 2 Department of Oncology, Hebei General Hospital, Shijiazhuang 050051, Hebei, P.R. China; 3 Department of Anesthesiology, Fourth Hospital of Hebei Medical University, Shijiazhuang 050011, Hebei, P.R. China

**Keywords:** Tsc22d3, morphine tolerance, iron death, GPX4, apoptosis

## Abstract

Background: Morphine tolerance refers to gradual reduction in response to drug with continuous or repeated use of morphine, requiring higher doses to achieve same effect.

Methods: The morphine tolerance dataset GSE7762 profiles, obtained from gene expression omnibus (GEO) database, were used to identify differentially expressed genes (DEGs). Weighted Gene Co-expression Network Analysis (WGCNA) was applied to explore core modules of DEGs related to morphine tolerance. Core genes were input into Comparative Toxicogenomics Database (CTD). Animal experiments were performed to validate role of Tsc22d3 in morphine tolerance and its relationship with ferroptosis-related pathway.

Results: 500 DEGs were identified. DEGs were primarily enriched in negative regulation of brain development, neuronal apoptosis processes, and neurosystem development. Core gene was identified as Tsc22d3. Tsc22d3 gene-associated miRNAs were mmu-miR-196b-5p and mmu-miR-196a-5p. Compared to Non-morphine tolerant group, Tsc22d3 expression was significantly upregulated in Morphine tolerant group. Tsc22d3 expression was upregulated in Morphine tolerant+Tsc22d3_OE, expression of HIF-1alpha, GSH, GPX4 in GPX4 ferroptosis-related pathway showed a more pronounced decrease. As Tsc22d3 expression was downregulated in Morphine tolerant+Tsc22d3_KO, expression of HIF-1alpha, GSH, GPX4 in GPX4 ferroptosis-related pathway exhibited a more pronounced increase. Upregulation of Tsc22d3 in Morphine tolerant+Tsc22d3_OE led to a more pronounced increase in expression of apoptosis proteins (P53, Caspase-3, Bax, SMAC, FAS). The expression of inflammatory factors (IL6, TNF-alpha, CXCL1, CXCL2) showed a more pronounced increase with upregulated Tsc22d3 expression in Morphine tolerant+Tsc22d3_OE.

Conclusions: Tsc22d3 is highly expressed in brain tissue of morphine-tolerant mice, activating ferroptosis pathway, enhancing apoptosis, promoting inflammatory responses in brain cells.

## INTRODUCTION

Morphine tolerance refers to the gradual reduction in the responsiveness to the drug with continuous or repeated use of morphine, requiring higher doses to achieve the same effect [1]. Morphine tolerance is relatively common in patients undergoing long-term morphine treatment, especially in those with chronic pain and cancer patients who use morphine for an extended period [2]. However, individual responses to morphine may vary, and not all individuals develop significant tolerance. The molecular mechanisms underlying morphine tolerance are not fully understood, but research suggests that it may be associated with various factors, including: Genetic Factors: Individual responses to morphine may be influenced by genetic factors [3]. Chromosomal Abnormalities: Changes at the chromosomal level may be linked to morphine tolerance [4]. Gene Fusion: Gene fusion events could lead to changes in gene expression, affecting tolerance to morphine. The etiology of morphine tolerance remains unclear and may involve genetic factors, chromosomal abnormalities, gene fusion, and other factors. Therefore, in-depth research into the molecular mechanisms of morphine tolerance is particularly important.

Bioinformatics technology is an interdisciplinary field that combines biology and computer science, utilizing computational and information technologies to process, analyze, and interpret biological data [5]. Bioinformatics technology has found widespread applications in various fields such as medicine, agriculture, and environmental science, driving the development of interdisciplinary research integration [6]. Bioinformatics provides the foundation for personalized medicine by analyzing individual genetic information, leading to a better understanding of disease mechanisms, and supporting the development of personalized treatment plans [7, 8]. This study employed bioinformatics technology to uncover genes, specifically Tsc22d3, associated with morphine tolerance.

The Tsc22d3 protein performs multiple functions within the cell. It is considered a transcription factor, participating in the regulation of gene transcription processes [9]. Additionally, Tsc22d3 is associated with processes such as inflammation, immune regulation, and cell survival. The expression of Tsc22d3 is also linked to cell survival and apoptosis [10]. Some studies suggest that Tsc22d3 may play a regulatory role in the cell’s lifecycle and death processes. However, the current understanding of the relationship between Tsc22d3 and morphine tolerance is not clear [11].

Therefore, this study aims to utilize bioinformatics technology and animal experiments to identify core genes associated with morphine tolerance compared to normal tissues. Enrichment analysis and pathway analysis will be conducted. The significant role of Tsc22d3 in morphine tolerance will be validated using public datasets, and animal experiments will be employed for further verification.

## RESULTS

### Differential gene analysis

**Figure 1:**
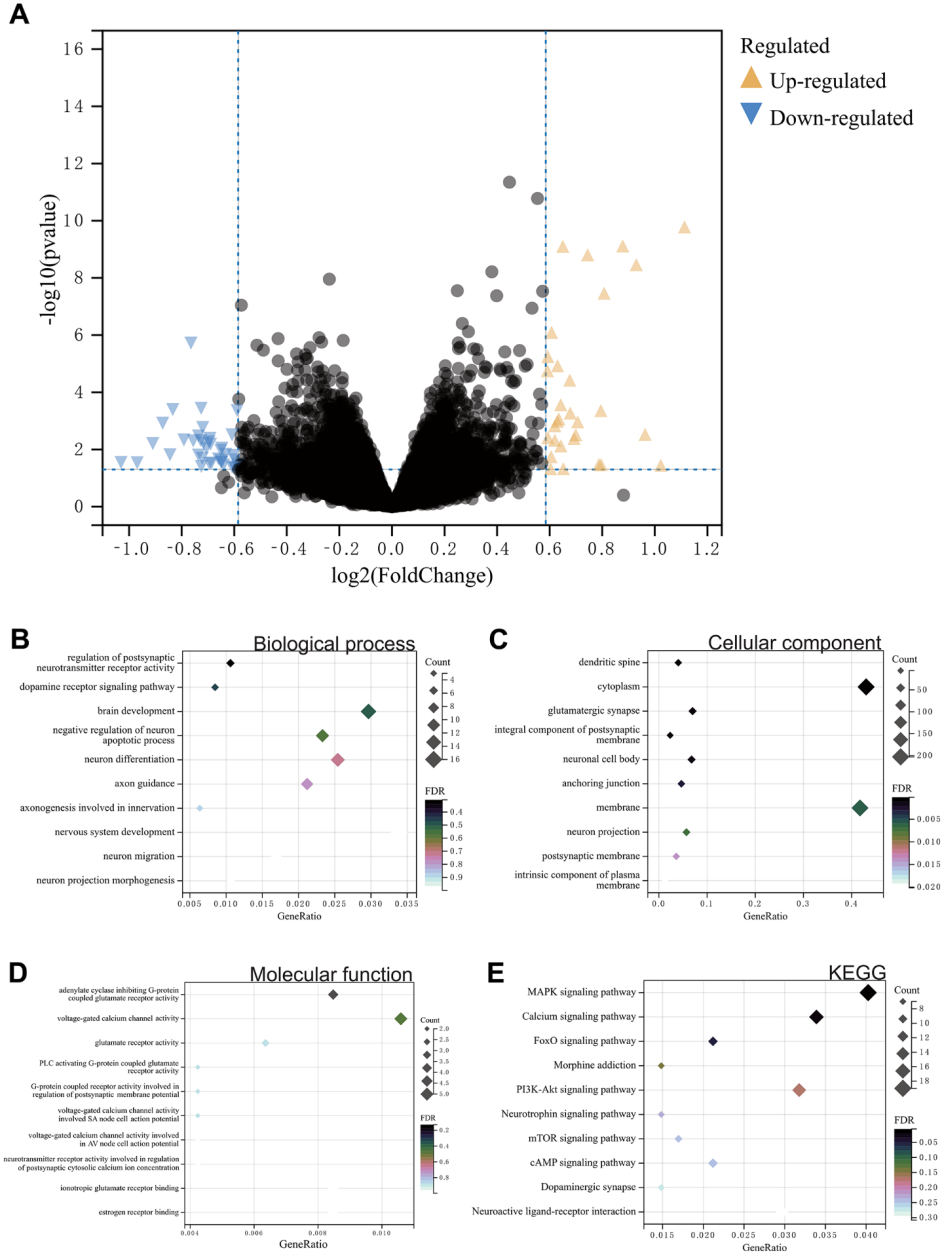
(**A**) Differential gene analysis. A total of 500 DEGs. (**B**–**E**) Results of GOKEGG enrichment analysis of DEGs. (**B**) Biological process analysis. (**C**) Cellular component analysis. (**D**) Molecular function analysis. (**E**) Results of KEGG enrichment analysis.

In this study, using the predefined cutoff values, we identified differentially expressed genes (DEGs) from the gene expression matrix of the GSE7762 dataset, resulting in a total of 500 DEGs (Figure 1A).

### Functional enrichment analysis

### GO and KEGG analysis of DEGs

Functional enrichment analysis, including GO and KEGG analysis, was performed on these differentially expressed genes. According to the GO analysis results, in Biological Process (BP) analysis, they were mainly enriched in negative regulation of brain development, neuronal apoptosis processes, and regulation of nervous system development (Figure 1B). In Cellular Component (CC) analysis, they were mainly enriched in neuronal projection and neuronal cell body (Figure 1C). In Molecular Function (MF) analysis, they were concentrated in the regulation of postsynaptic cytoplasmic calcium ion concentration involving neurotransmitter receptor activity and regulation of postsynaptic membrane potential by G-protein coupled receptor activity (Figure 1D). In KEGG analysis, they were mainly enriched in opioid addiction, dopaminergic synapse, neuroactive ligand-receptor interaction, and MAPK signaling pathway (Figure 1E).

### Metascape enrichment analysis

**Figure 2:**
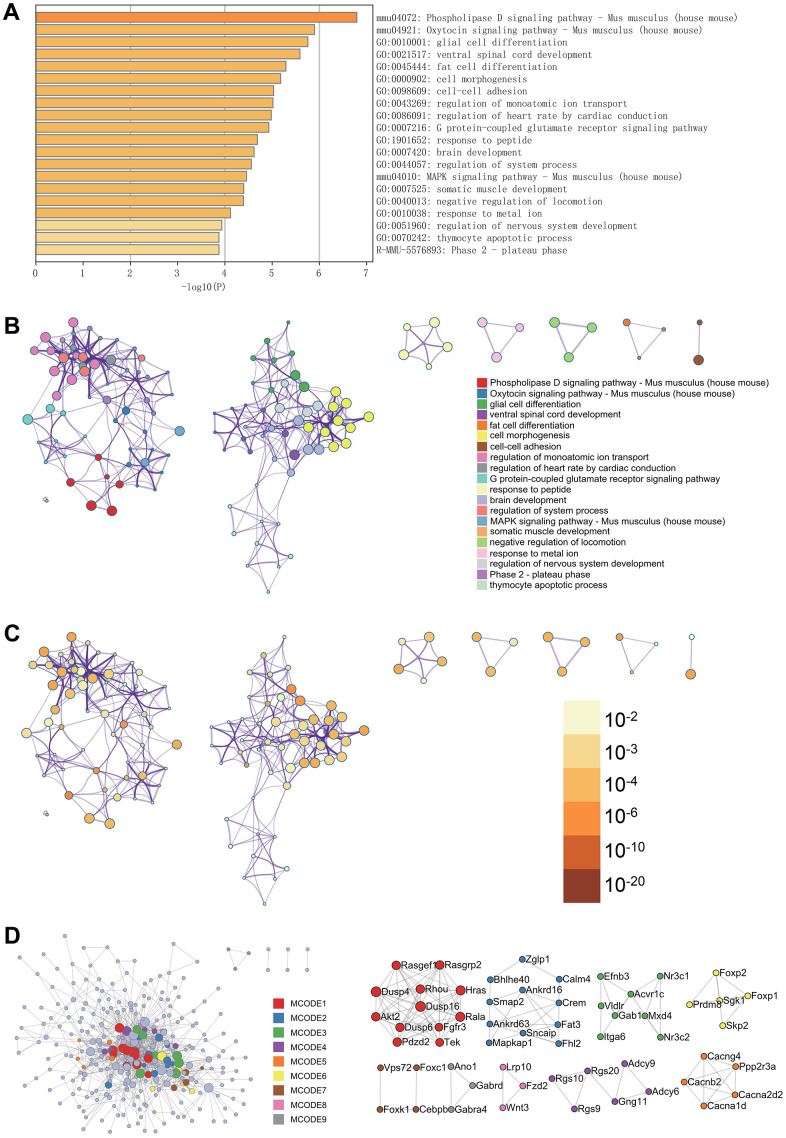
**Metascape enrichment analysis.** (**A**) Bar graph of enriched terms across input gene lists, colored by p-values. (**B**) Network of enriched terms: colored by cluster ID, where nodes that share the same cluster ID are typically close to each other. (**C**) colored by p-value, where terms containing more genes tend to have a more significant p-value. (**D**) Protein-protein interaction network. MCODE components identified in the gene lists.

In the Metascape enrichment projects, regulatory aspects of brain development, nervous system development, and the MAPK signaling pathway in mice were visible in the GO enrichment projects (Figure 2A). Additionally, we generated enrichment networks colored by enrichment terms and p-values (Figure 2B–2D), providing a visual representation of the associations and confidence levels of various enrichment projects.

### WGCNA

**Figure 3:**
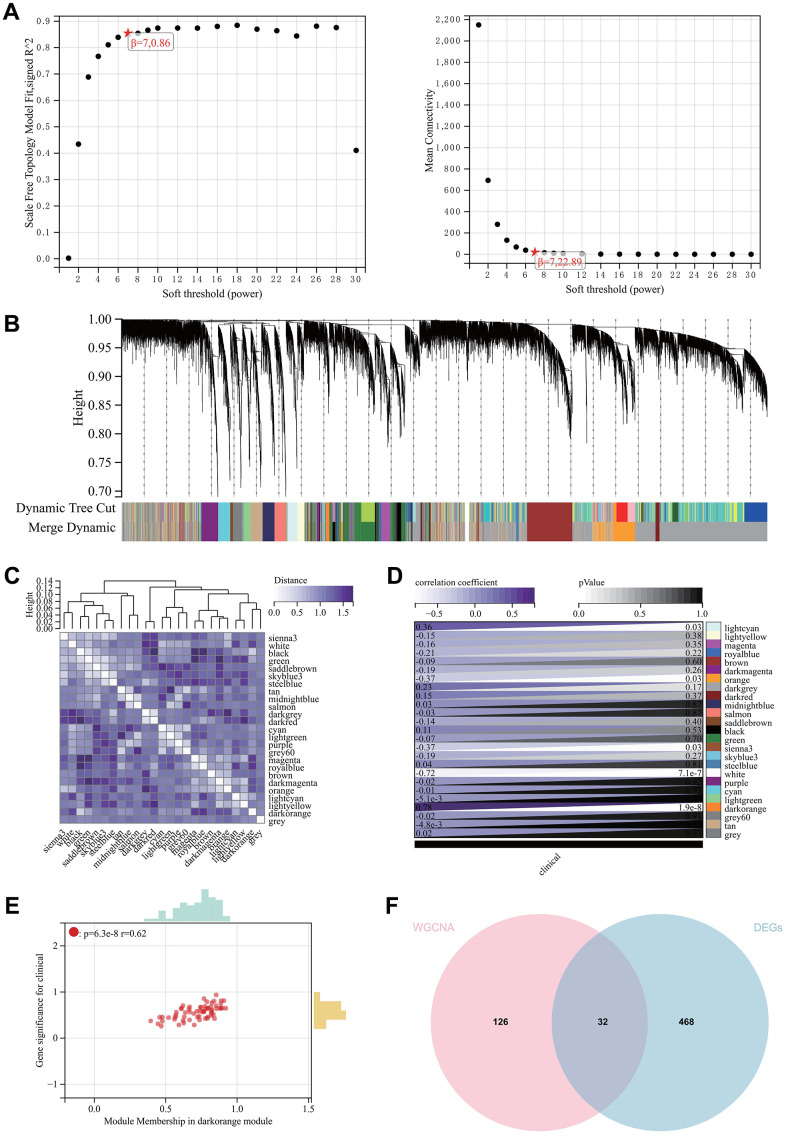
**WGCNA.** (**A**) β = 6,0.86. β = 6, 22.89. (**B**, **C**) The hierarchical clustering tree of all genes was constructed, and 25 important modules were generated. (**D**) The heat map of correlation between modules and phenotypes. (**E**) The scatter map of correlation between GS and MM of related hub genes. (**F**) The DEGs screened by WGCNA and DEGs were used to obtain Venn map. 32 intersection genes were obtained.

The selection of the soft-thresholding power is a crucial step in WGCNA analysis. The soft-thresholding power for network topology analysis was set to 7 (Figure 3A). A hierarchical clustering tree was constructed for all genes, resulting in 25 modules (Figure 3B). Interaction between important modules was analyzed (Figure 3C), and a heatmap of module-phenotype correlation (Figure 3D) and a scatter plot of the correlation between GS and MM of relevant hub genes (Figure 3E) were generated.

We also created a Venn diagram by intersecting genes selected by WGCNA with DEGs for further analysis (Figure 3F).

### Protein-protein interaction (PPI) network construction and analysis

**Figure 4:**
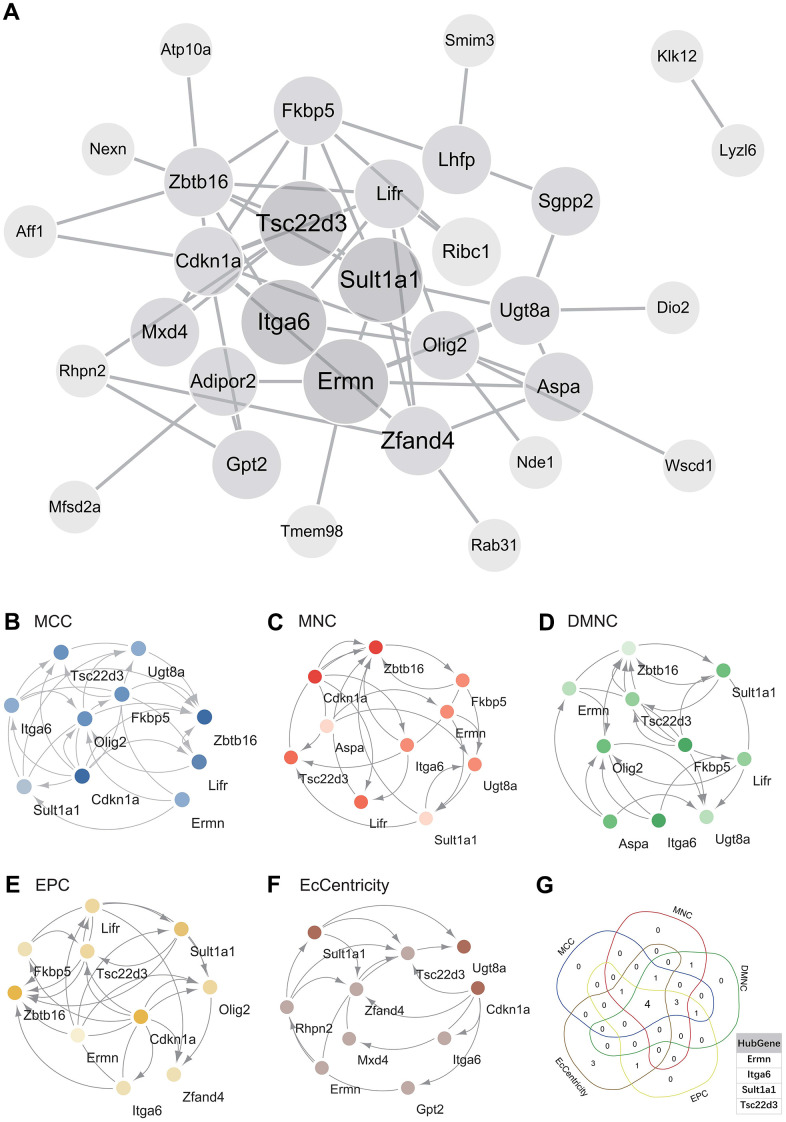
**Construction and analysis of protein-protein interaction (PPI) networks.** (**A**) Construct the PPI network of DEGs using STRING online database and utilize Cytoscape software for analysis. (**B**) MCC was used to identify the central gene. (**C**) MNC was used to identify the central gene. (**D**) DMNC was used to identify the central gene. (**E**) EPC was used to identify the central gene. (**F**) EcCentricity was used to identify the central gene. (**G**) Core genes (Tsc22d3, Itga6, Ermn, Sult1a1) were obtained by merging using Venn diagrams.

The PPI network of DEGs was constructed using the STRING online database and analyzed using Cytoscape software (Figure 4A). Central genes were identified using five different algorithms (MCC, MNC, DMNC, EPC, EcCentricity) (Figure 4B–4F). The Venn diagram was used to obtain the intersection (Figure 4G), resulting in four core genes (Tsc22d3, Itga6, Ermn, Sult1a1).

### Gene expression heatmap

**Figure 5:**
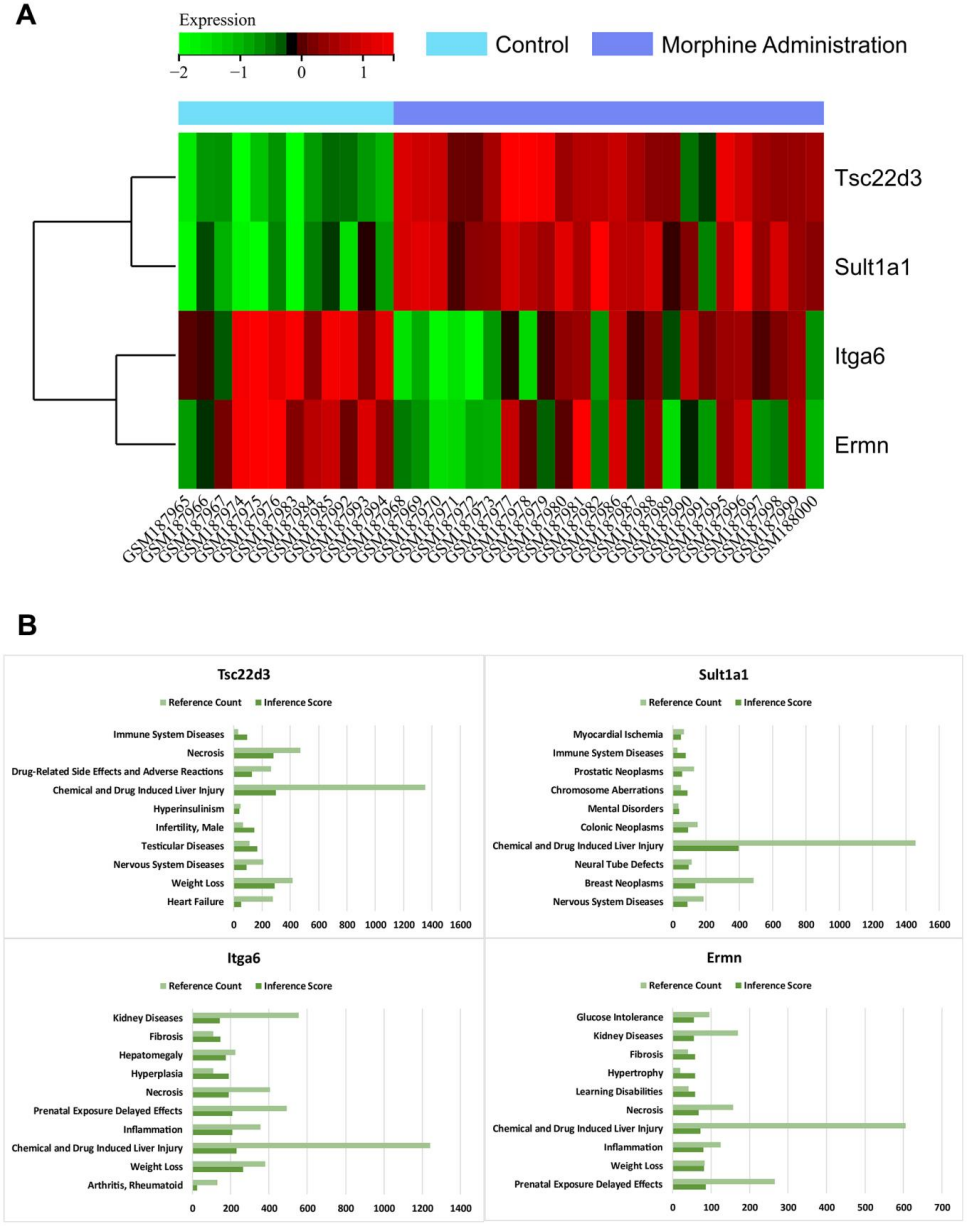
**Heat map of gene expression.** (**A**) Heat map of the core gene in the gene expression matrix of the data sets GSE7762. (**B**) CTD analysis. Core genes (Tsc22d3, Itga6, Ermn, Sult1a1) are associated with immune system diseases, drug-related side effects and adverse reactions, chemical and drug-induced liver injury, neurological diseases, heart failure, mental disorders, and neural tube defects.

We visualized the expression levels of core genes in the gene expression matrix of the GSE7762 dataset and created a heatmap (Figure 5A). We observed that core genes (Tsc22d3, Sult1a1) were highly expressed in brain tissue samples injected with morphine, while they were lowly expressed in normal samples, indicating significant differences. Based on these results, we speculate that these core genes may play a regulatory role in morphine tolerance after injection.

### CTD analysis

In this study, we input the hub gene list into the CTD website to identify diseases related to the core genes, enhancing our understanding of gene-disease associations. We found that the four core genes (Tsc22d3, Itga6, Ermn, Sult1a1) were associated with immune system diseases, drug-related side effects and adverse reactions, chemical and drug-induced liver injury, neurological disorders, heart failure, mental disorders, and neural tube defects (Figure 5B).

### Prediction and functional annotation of miRNAs associated with hub genes

In this study, we input the hub gene list into TargetScan to identify relevant miRNAs, enhancing our understanding of gene expression regulation (Table 1). We found that the relevant miRNAs for the Tsc22d3 gene were mmu-miR-196b-5p and mmu-miR-196a-5p; for the Itga6 gene, it was mmu-miR-126a-3p.1; and for the Ermn gene, it was mmu-miR-582-5p, mmu-miR-219a-2-3p, and mmu-miR-496a-3p.2.

**Table 1:** A summary of miRNAs that regulate hub genes.

	**Gene**	**MIRNA**		
**1**	**Tsc22d3**	mmu-miR-196b-5p	mmu-miR-196a-5p	
**2**	**Itga6**	mmu-miR-126a-3p.1		
**3**	**Ermn**	mmu-miR-582-5p	mmu-miR-219a-2-3p	mmu-miR-496a-3p.2
**4**	**Sult1a1**	None		

### Impact of Tsc22d3 on iron death-related pathway protein expression in morphine tolerance

**Figure 6:**
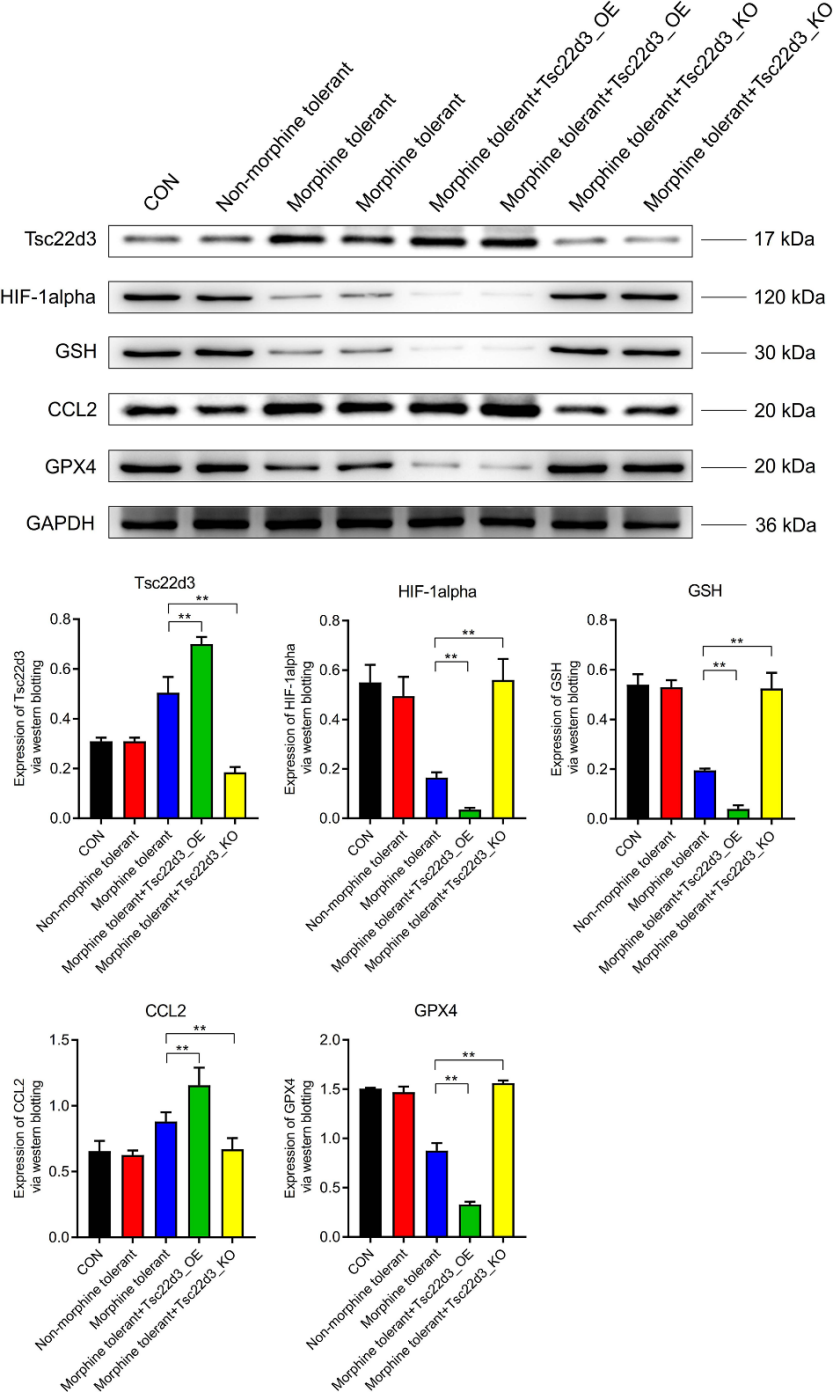
Effect of Tsc22d3 on iron death-related pathway protein expression in morphine tolerance.

The expression of Tsc22d3 was not significantly different between the CON group and the Non-morphine tolerant group. Compared to the Non-morphine tolerant group, the expression of Tsc22d3 was significantly upregulated in the Morphine tolerant group. Compared to the Morphine tolerant group, the expression of Tsc22d3 was further upregulated in the Morphine tolerant+Tsc22d3_OE group. Compared to the Morphine tolerant group, the expression of Tsc22d3 was significantly downregulated in the Morphine tolerant+Tsc22d3_KO group. The key node proteins (HIF-1alpha, GSH, CCL2, GPX4) in the GPX4 iron death-related pathway did not show significant differences in expression between the CON group and the Non-morphine tolerant group. Compared to the Non-morphine tolerant group, the expression of key node proteins (HIF-1alpha, GSH, GPX4) in the GPX4 iron death-related pathway was significantly downregulated in the Morphine tolerant group. Compared to the Non-morphine tolerant group, the expression of the key node protein (CCL2) in the GPX4 iron death-related pathway was significantly upregulated in the Morphine tolerant group. With the upregulation of Tsc22d3 expression in Morphine tolerant+Tsc22d3_OE, the expression of key node proteins (HIF-1alpha, GSH, GPX4) in the GPX4 iron death-related pathway further decreased significantly. With the upregulation of Tsc22d3 expression in Morphine tolerant+Tsc22d3_OE, the expression of the key node protein (CCL2) in the GPX4 iron death-related pathway further increased significantly. With the downregulation of Tsc22d3 expression in Morphine tolerant+Tsc22d3_KO, the expression of key node proteins (HIF-1alpha, GSH, GPX4) in the GPX4 iron death-related pathway further increased significantly. With the downregulation of Tsc22d3 expression in Morphine tolerant+Tsc22d3_KO, the expression of the key node protein (CCL2) in the GPX4 iron death-related pathway further decreased significantly (Figure 6).

### Tsc22d3 promotes apoptosis of brain cells in morphine tolerance

**Figure 7:**
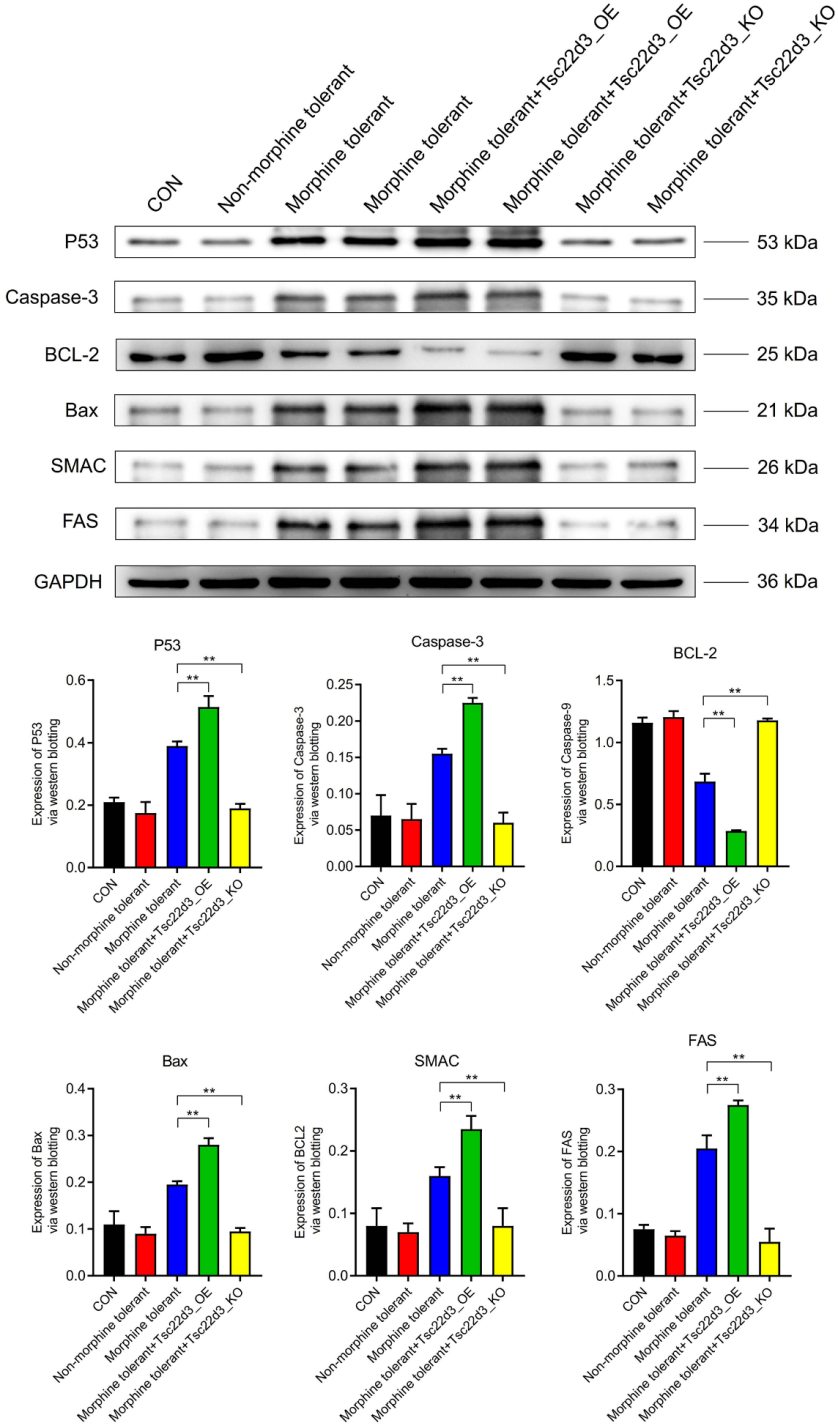
Tsc22d3 promotes apoptosis of brain cells in morphine tolerance.

Compared to the CON group, the expression of apoptosis proteins (P53, Caspase-3, BCL-2, Bax, SMAC, FAS) in the Non-morphine tolerant group was not significantly different. Compared to the Non-morphine tolerant group, the expression of apoptosis proteins (P53, Caspase-3, Bax, SMAC, FAS) was significantly upregulated in the Morphine tolerant group. Compared to the Non-morphine tolerant group, the expression of the anti-apoptotic protein (BCL-2) was significantly downregulated in the Morphine tolerant group. With the upregulation of Tsc22d3 expression in Morphine tolerant+Tsc22d3_OE, the expression of apoptosis proteins (P53, Caspase-3, Bax, SMAC, FAS) further increased significantly. With the upregulation of Tsc22d3 expression in Morphine tolerant+Tsc22d3_OE, the expression of the anti-apoptotic protein (BCL-2) further decreased in the Morphine tolerant group. With the downregulation of Tsc22d3 expression in Morphine tolerant+Tsc22d3_KO, the expression of apoptosis proteins (P53, Caspase-3, Bax, SMAC, FAS) further decreased significantly. With the downregulation of Tsc22d3 expression in Morphine tolerant+Tsc22d3_KO, the expression of the anti-apoptotic protein (BCL-2) further increased in the Morphine tolerant group (Figure 7).

### Tsc22d3 promotes inflammatory response of brain cells in morphine tolerance

**Figure 8:**
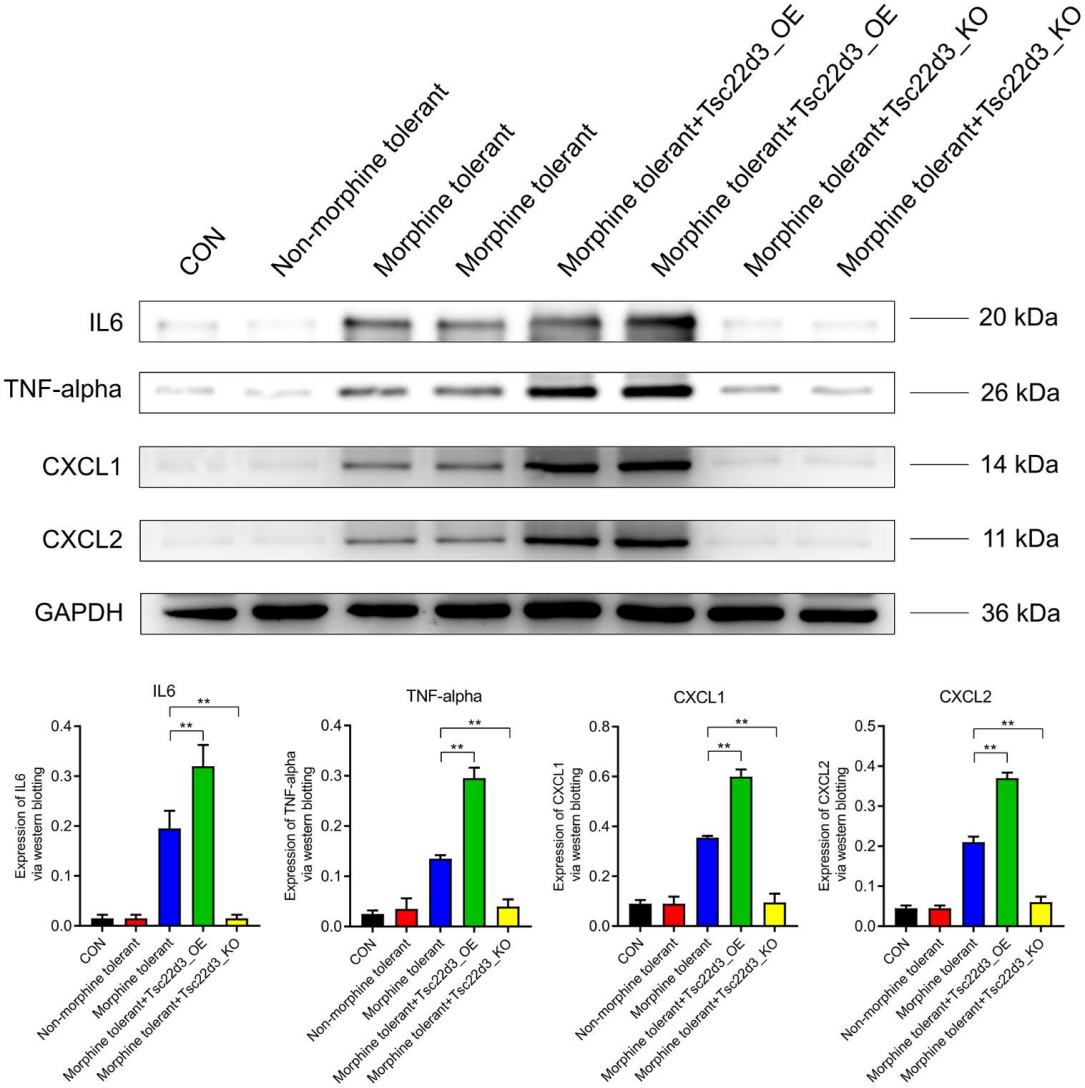
Tsc22d3 promotes inflammation in brain cells during morphine tolerance.

Compared to the CON group, the expression of inflammatory factors (IL6, TNF-alpha, CXCL1, CXCL2) in the Non-morphine tolerant group did not show significant differences. However, when compared to the Non-morphine tolerant group, the expression of inflammatory factors (IL6, TNF-alpha, CXCL1, CXCL2) was significantly upregulated in the Morphine tolerant group. With the upregulation of Tsc22d3 expression in Morphine tolerant+Tsc22d3_OE, the expression of inflammatory factors (IL6, TNF-alpha, CXCL1, CXCL2) further increased significantly. Conversely, with the downregulation of Tsc22d3 expression in Morphine tolerant+Tsc22d3_KO, the expression of inflammatory factors (IL6, TNF-alpha, CXCL1, CXCL2) further decreased significantly (Figure 8).

## DISCUSSION

Morphine tolerance leads patients to require higher doses to maintain the same therapeutic effect, potentially causing a gradual reduction in the pain-relieving effects of morphine [2]. Tolerance may prompt patients to seek higher doses to achieve the desired effects, increasing the risk of morphine abuse and addiction. In-depth exploration of the molecular mechanisms of morphine tolerance is crucial for research on targeted drugs [12]. The main findings of this study indicate that Tsc22d3 is highly expressed in morphine-tolerant brain tissue, activating iron death-related pathway proteins, inducing apoptosis, and triggering inflammatory responses in brain cells.

Morphine tolerance typically involves various factors such as neural adaptation, changes in signaling pathways, and alterations in drug metabolism. Tsc22d3 may influence certain pathological processes related to morphine tolerance through its role in inflammation and immune regulation. For example, if the expression of Tsc22d3 is regulated in certain cells or tissues, it could impact inflammation levels, indirectly affecting neural adaptation and signaling pathways, thus influencing the effects of morphine and the development of tolerance [13].

There is a certain relationship between Tsc22d3 and apoptosis [14]. Apoptosis is a programmed cell death and is one of the essential mechanisms for maintaining normal development of tissues and organs, as well as the homeostasis of the internal environment [15]. Tsc22d3 may influence the survival and death of cells by regulating genes associated with apoptosis. It may participate in the transcriptional regulation of genes as a transcription factor, thereby affecting the activation of the apoptosis pathway [16]. The anti-inflammatory and immune-regulatory effects of Tsc22d3 may be related to its regulation during the apoptosis process. Inflammation and immune processes are often interconnected with cell apoptosis, and the regulation by Tsc22d3 may intervene in the control of these processes, thereby affecting the survival and death of cells [17]. Tsc22d3 may exhibit different functions in various cell types and environments, and its molecular mechanisms related to apoptosis may involve multiple pathways. The expression of Tsc22d3 can impact the function of immune cells, such as macrophages and T cells [18].

Tsc22d3 plays a significant role in suppressing inflammatory responses, potentially influencing various molecular mechanisms related to inflammation through multiple pathways [19]. A deeper understanding of this field contributes to revealing the specific role of Tsc22d3 in immune regulation and the inflammatory process, providing new targets and strategies for the treatment of inflammatory diseases [20]. Long-term use of morphine may lead to the occurrence of neuroadaptation, where neurons gradually become less responsive to the drug. This neuroadaptation is an important mechanism of morphine tolerance, where changes in cell signaling pathways result in a reduced response to the effects of morphine. Morphine-induced apoptosis: Some studies suggest that morphine may have pro-apoptotic effects. This pro-apoptotic effect may be achieved through multiple pathways, including the regulation of apoptosis-related proteins and the impact on mitochondrial function. Bcl-2 family: The Bcl-2 family is a protein family closely associated with the regulation of cell apoptosis. Morphine may influence cell survival and apoptosis by regulating the expression of Bcl-2 family members [21]. Some studies have found that morphine may inhibit apoptosis by reducing the expression of the anti-apoptotic protein Bcl-2 or increasing the expression of the pro-apoptotic protein Bax. Inflammation and oxidative stress: Long-term morphine use is also associated with inflammation and oxidative stress processes, which may affect cell survival and apoptosis [22]. Chronic inflammation and oxidative stress may be important factors in morphine tolerance and associated cellular damage [10, 23, 24].

There is a close relationship between morphine tolerance and inflammation, and prolonged use of morphine may induce inflammatory responses, while the inflammatory process can also impact the pharmacological effects of morphine. Inflammation and Morphine Tolerance: Prolonged use of morphine may lead to the body generating an inflammatory response [25]. The pharmacological effects of morphine are related to the interaction between neurons and immune cells, and this interaction may result in the occurrence of inflammation. Chronic inflammation may be associated with the development of morphine tolerance, causing a gradual reduction in the patient’s response to morphine [26].

Neuroadaptation and Inflammation: The formation of morphine tolerance may be related to neuroadaptation and inflammation in neurons. Long-term use of morphine may lead to adaptive changes in neurons, where the activity and sensitivity of neurons to morphine decrease, requiring higher doses to produce the same effect [27]. Toll-Like Receptors (TLR) and Inflammatory Response: Some studies suggest that morphine may participate in inflammatory responses by affecting Toll-like receptor (TLR) signaling pathways [2]. TLRs are a class of immune sensing receptors that play a crucial role in the inflammatory process [28]. Morphine may regulate inflammatory responses by influencing TLR signaling pathways, thereby affecting the pharmacological effects and tolerance of morphine [29]. Activation of NF-κB Pathway: Inflammatory responses are often associated with the activation of the NF-κB (nuclear factor kappa B) pathway. The use of morphine may impact inflammatory responses by modulating the activation state of the NF-κB pathway [30]. NF-κB is a crucial transcription factor that regulates the expression of multiple inflammation-related genes. Involvement of Immune Cells: The effects of morphine are not limited to neurons but also involve immune cells. Prolonged use of morphine may affect the function of immune cells, leading to immune suppression or the occurrence of inflammation, thereby influencing the effectiveness of morphine [31].

This study aims to further investigate the role of Tsc22d3 in opioid tolerance using animal models. It will explore how the expression or activity of Tsc22d3 influences cellular apoptosis and *in vivo* inflammatory responses. By delving deeper into the comprehensive understanding of Tsc22d3’s involvement in opioid tolerance, this research sets the groundwork for the development of novel therapeutic interventions in this field. While this study conducted a rigorous analysis, there are still some limitations. The research did not include clinical specimen experiments to further validate its findings, primarily due to difficulties in obtaining brain tissues from patients. In future studies, it is essential to delve into this aspect for a more comprehensive exploration.

### Research outlook

This study holds promising potential for clinical applications. Further research confirming Tsc22d3 as a key regulatory factor in opioid tolerance could lead to the development of related drugs or treatment strategies to help patients cope with opioid tolerance more effectively.

## CONCLUSIONS

In summary, Tsc22d3 is highly expressed in the brain tissues of morphine-tolerant mice, subsequently activating the iron death pathway and enhancing apoptosis and inflammatory responses in brain cells. Future research directions will further explore the regulatory mechanisms of Tsc22d3 in opioid tolerance, develop therapeutic approaches targeting this gene, and investigate the clinical application prospects, enriching the content and potential applications of this study.

## MATERIALS AND METHODS

### Morphine tolerance dataset

The morphine tolerance dataset GSE7762 profile was downloaded from the gene expression omnibus (GEO) database (http://www.ncbi.nlm.nih.gov/geo/), generated from GPL1261 (https://www.ncbi.nlm.nih.gov/geo/query/acc.cgi?acc=GSE7762), to identify differentially expressed genes (DEGs) in the brain tissue associated with morphine tolerance.

### DEGs selection

The R package “limma” was used for probe summarization and background correction of the gene expression matrix from GSE7762. The Benjamini-Hochberg method was employed to adjust the raw p-values. The fold change (FC) was calculated using the false discovery rate (FDR). The cutoff criterion for DEGs was set at P < 0.05. A volcano plot was generated to visualize the DEGs.

### Weighted gene co-expression network analysis (WGCNA)

Initially, the Median Absolute Deviation (MAD) for each gene was calculated using the gene expression matrix of GSE7762. The bottom 50% of genes with the smallest MAD values were excluded. The “goodSamplesGenes” method from the R package WGCNA was utilized to remove outlier genes and samples. To categorize genes with similar expression profiles into gene modules, hierarchical average linkage clustering based on TOM-based dissimilarity was performed, with a minimum module size (number of genes) set at 30. The sensitivity was set at 3. For further module analysis, dissimilarity of module characteristic genes was calculated, and a cutting line was chosen for module dendrogram, merging some modules. Additionally, modules with a distance less than 0.25 were merged. It is noteworthy that the grey module is considered a collection of genes that cannot be assigned to any module.

### Construction and analysis of protein-protein interaction (PPI) network

The STRING database (http://string-db.org/) aims to collect, score, and integrate all publicly available protein-protein interaction information sources and supplement them through computational predictions. In this study, the list of differentially expressed genes (DEGs) was input into the STRING database to construct a predicted core gene PPI network (confidence > 0.4). Cytoscape software provides biologists with tools for biological network analysis and two-dimensional (2D) visualization. In this study, the PPI network formed by the STRING database was visualized and the core genes were predicted using Cytoscape software. The PPI network was imported into Cytoscape, and the most relevant modules were identified using MCODE. Additionally, five algorithms (MCC, MNC, DMNC, EPC, EcCentricity) were employed to calculate the top ten genes with the best relevance, and the intersection was taken. The resulting core gene list was visualized and exported after visualization.

### Functional enrichment analysis

Gene Ontology (GO) and Kyoto Encyclopedia of Genes and Genomes (KEGG) analyses are computational methods for assessing gene function and biological pathways. In this study, the list of differentially expressed genes selected by the Venn diagram was input into the KEGG REST API (https://www.kegg.jp/kegg/rest/keggapi.html) to obtain the latest gene annotations for KEGG pathways. This was used as a background for mapping genes to the background set. Enrichment analysis was performed using the R package clusterProfiler (version 3.14.3) to obtain results of gene set enrichment. Additionally, GO annotations for genes from the org.Hs.eg.db package (version 3.1.0) were used as a background, with a minimum gene set size of 5, a maximum gene set size of 5000, and criteria of P-value < 0.05 and FDR < 0.25 considered statistically significant.

Furthermore, the Metascape database provides comprehensive gene list annotation and analysis resources with visualization capabilities. Metascape (http://metascape.org/gp/index.html) was used for functional enrichment analysis of the aforementioned list of differentially expressed genes, and the results were exported.

### Gene expression heatmap

The R package heatmap was utilized to create a heatmap of the expression levels of core genes identified in the PPI network in the gene expression matrix of GSE7762. This visualization depicts the expression differences between brain tissues injected with morphine and normal brain tissues.

### CTD Analysis

The Comparative Toxicogenomics Database (CTD) integrates a vast amount of data on interactions between chemicals, genes, phenotypes, and diseases, providing significant convenience for studying disease-related environmental exposure factors and potential drug mechanisms. The core genes were input into the CTD website, and the most relevant diseases associated with the core genes were identified. Radar plots depicting the expression differences for each gene were created using Excel.

### miRNA

TargetScan (www.targetscan.org) is an online database used for predicting and analyzing miRNA and their target genes. In our study, TargetScan was employed to screen miRNAs regulating central differentially expressed genes (DEGs).

### Animal experiments

Fifty C57BL/6J mice (6-8 weeks old) were purchased from Sibeifu (Beijing) Biotechnology Co., Ltd., acclimated for one week, and randomly divided into five groups, each consisting of 10 mice.

Control group (CON): Injected with physiological saline;

Non-morphine tolerant group (Non-morphine tolerant group): Single-dose administration to mice (20 mg/kg, s.c.);

Morphine tolerant group (Morphine tolerant group): Multiple-dose administration to mice (10 ~ 40 mg/kg, three times a day, for 5 days);

Morphine tolerant + Tsc22d3_KO group (Morphine tolerant + Tsc22d3_KO group): Multiple-dose administration to mice (10 ~ 40 mg/kg, three times a day, for 5 days) with Tsc22d3 knocked down using siRNA technology. Plasmids were constructed and injected into mice via tail vein injection.

Morphine tolerant + Tsc22d3_OE group (Morphine tolerant + Tsc22d3_OE group): Multiple-dose administration to mice (10 ~ 40 mg/kg, three times a day, for 5 days) with Tsc22d3 overexpressed. Plasmids were constructed and injected into mice via tail vein injection.

### Detection of iron death-related pathway proteins

Brain tissue was isolated, and protein extraction was performed. The tissue chunks were washed 2-3 times with pre-chilled PBS to remove blood stains. They were then cut into small pieces and placed in a homogenizer tube. A 4mm homogenization bead was added, along with lysis buffer (containing various protease inhibitors) at 10 times the tissue volume. Homogenization was performed, and the homogenization tube was taken out and placed on ice for 30 minutes. The supernatant collected after centrifugation at 12000rpm, 4° C, for 10 minutes was considered the total protein solution. The BCA protein assay kit (Invitrogen) was used to determine the protein concentration. Protein denaturation was performed by adding 4:1 protein solution to 5× reducing protein loading buffer, boiling for 15 minutes in a water bath, and storing at -20° C. Different concentration separation gels were prepared based on experimental requirements. The gel was poured after adding TEMED, and a comb was inserted into the gel. Gel electrophoresis was conducted at a constant voltage of 200V for approximately 30 minutes. Once complete, bromophenol blue reached about 1cm from the bottom, and the electrophoresis was terminated for membrane transfer.

For membrane transfer, activated PVDF (0.45um) membrane was used after activation with methanol for 2 minutes. The gel was carefully peeled off and placed on filter paper, and the PVDF membrane was applied to the gel. Three pieces of filter paper were placed on top of the membrane, and any remaining water was removed. The transfer conditions were set as a constant current of 300mA for 30 minutes with the transfer equipment placed in ice water to cool during the process. The transferred membrane was blocked with 5% skimmed milk for 1 hour and then incubated with primary antibodies. Visualization of immune reaction bands was achieved through chemiluminescence using horseradish peroxidase-conjugated IgG secondary antibodies. Band density was measured using a densitometry method.

### Data availability

The datasets generated during and/or analyzed during the current study are available from the corresponding author on reasonable request.
